# Administration of broadly neutralizing anti-HIV-1 antibodies at ART initiation maintains long-term CD8^+^ T cell immunity

**DOI:** 10.1038/s41467-022-34171-2

**Published:** 2022-10-29

**Authors:** Miriam Rosás-Umbert, Jesper D. Gunst, Marie H. Pahus, Rikke Olesen, Mariane Schleimann, Paul W. Denton, Victor Ramos, Adam Ward, Natalie N. Kinloch, Dennis C. Copertino, Tuixent Escribà, Anuska Llano, Zabrina L. Brumme, R. Brad Jones, Beatriz Mothe, Christian Brander, Julie Fox, Michel C. Nussenzweig, Sarah Fidler, Marina Caskey, Martin Tolstrup, Ole S. Søgaard

**Affiliations:** 1 Department of Clinical Medicine, Aarhus University, Aarhus, Denmark; 2 Department of Infectious Diseases, Aarhus University Hospital, Aarhus, Denmark; 3 Department of Biology, University of Nebraska at Omaha, Omaha, NE USA; 4 Laboratory of Molecular Immunology, The Rockefeller University, New York, NY USA; 5 Infectious Diseases Division, Department of Medicine, Weill Cornell Medical College, New York, NY USA; 6 Department of Microbiology and Immunology, Weill Cornell Graduate School of Medical Sciences, New York, NY USA; 7 Faculty of Health Sciences, Simon Fraser University, Burnaby, BC Canada; 8 British Columbia Centre for Excellence in HIV/AIDS, Vancouver, BC Canada; 9 IrsiCaixa, AIDS Research Institute, Institute for Health Science Research Germans Trias i Pujol (IGTP), Hospital Germans Trias I Pujol, Badalona, Spain; 10 CIBERINFEC, ISCIII, Madrid, Spain; 11 Centre for Health and Social Care Research (CESS), Faculty of Medicine, University of Vic—Central University of Catalonia (UVic—UCC), Vic, Barcelona, Spain; 12 Institució Catalana de Recerca i Estudis Avançats (ICREA), Barcelona, Spain; 13 Department of Genitourinary Medicine and Infectious Disease, Guys and St Thomas’ National Health Service Trust, London, UK; 14 Department of Genitourinary Medicine and Infectious Disease, The National Institute for Health Research Biomedical Research Centre, King’s College London, London, UK; 15 Howard Hughes Medical Institute, The Rockefeller University, New York, NY USA; 16 Department of Infectious Diseases, Imperial College London, London, UK; 17 The National Institute for Health Research, Imperial Biomedical Research Centre, London, UK

**Keywords:** HIV infections, Translational research

## Abstract

In simian-human immunodeficiency virus (SHIV)-infected non-human primates, broadly neutralizing antibodies (bNAbs) against the virus appear to stimulate T cell immunity. To determine whether this phenomenon also occurs in humans we measured HIV-1-specific cellular immunity longitudinally in individuals with HIV-1 starting antiviral therapy (ART) with or without adjunctive bNAb 3BNC117 treatment. Using the activation-induced marker (AIM) assay and interferon-γ release, we observe that frequencies of Pol- and Gag-specific CD8^+^ T cells, as well as Gag-induced interferon-γ responses, are significantly higher among individuals that received adjunctive 3BNC117 compared to ART-alone at 3 and 12 months after starting ART. The observed changes in cellular immunity were directly correlated to pre-treatment 3BNC117-sensitivity. Notably, increased HIV-1-specific immunity is associated with partial or complete ART-free virologic control during treatment interruption for up to 4 years. Our findings suggest that bNAb treatment at the time of ART initiation maintains HIV-1-specific CD8^+^ T cell responses that are associated with ART-free virologic control.

## Abstract (web summary)

Broadly neutralising anti-HIV-1 antibody (bNAb) administration in nonhuman primates has been shown to stimulate adaptive T cell-specific immunity, with infection prevention observed. In this work, the authors longitudinally analyse HIV-1 specific cellular immunity in HIV-1- infected individuals starting ART with or without adjunctive bNAb treatment.

## Introduction

Antiretroviral therapy (ART) effectively suppresses HIV-1 viral replication but ART does not eliminate infected cells^1,2^. Due to the integration of replication competent proviruses in long-lived CD4^+^ T cells, lifelong medication is needed to prevent disease progression. If individuals on treatment stop taking ART, viral replication resumes which generally leads to rapid viral rebound of plasma viremia within a few weeks^3^. Many strategies are being pursued to achieve a functional cure or complete virus eradication, including the administration of broadly neutralizing anti-HIV-1 antibodies (bNAbs)^4^.

bNAbs are able to neutralize free viral particles and block the infection of target cells by the binding of their variable domain region to HIV-1 envelope^5–8^. The administration of bNAbs has been shown to prevent infection in animal models^9–11^ and maintain viral suppression when administered during analytical treatment interruption (ATI) in humans^12,13^. In addition, bNAbs may have a vaccinal effect by stimulating adaptive T cell-specific immunity via immune complex formation leading to dendritic cell activation and enhanced antigen processing and presentation^14^. This effect has been shown to increase the chance of CD8^+^ T cell-mediated post-treatment control in macaques infected with chimeric simian and human immunodeficiency virus (SHIV). In a study by Nishimura et al., the induced CD8^+^ T cell responses following early administration of bNAbs in SHIV_AD8_-infected macaques contributed to virologic control which was observed in half of the bNAb-treated animals compared to none of the ART-treated control animals^15,16^. In clinical trials, bNAb administration has also been associated with increased T cell responses during ART interruption^12,14^. Whether this augmentation of virus-specific T cell immunity can also be induced with bNAb administration at the time of ART initiation remains to be determined. To address this question, we profiled HIV-1-specific T cell immune responses longitudinally and analyzed their relationship to ART-free virologic control in individuals newly diagnosed with HIV-1 starting ART with or without the potent bNAb 3BNC117 and with or without romidepsin (RMD)^17^.

## Results

### Sustained HIV-1 Gag-specific CD8^+^ T cell responses after bNAb administration

**Fig. 1:**
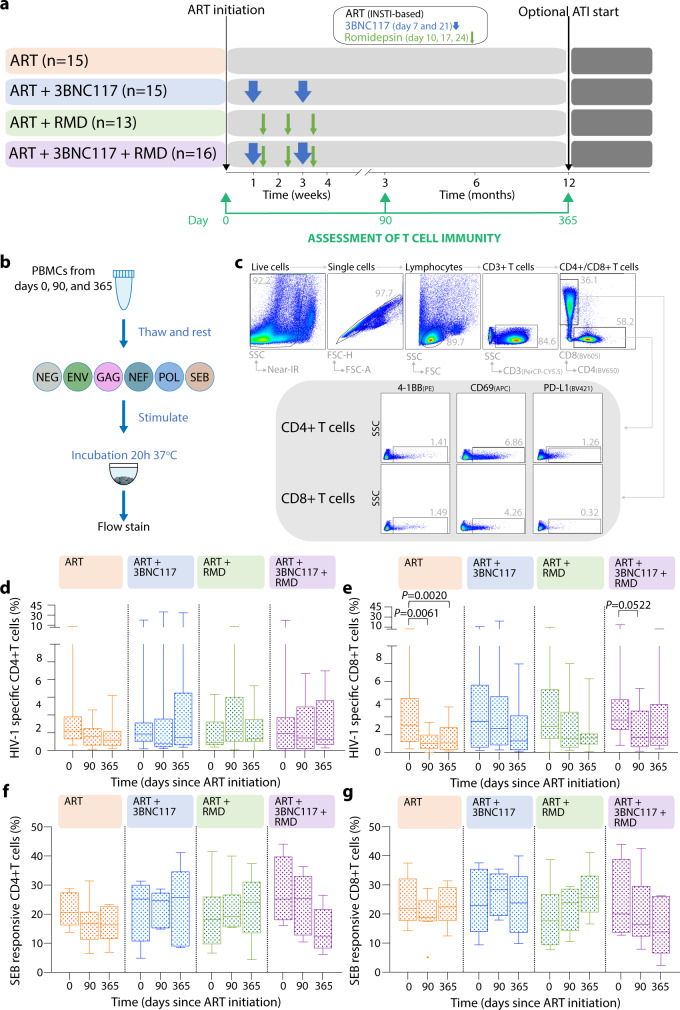
Evaluation of total HIV-specific T cell responses by AIM assay. **a** Study design and samples evaluated. **b** Representation of the activation-induced marker (AIM) protocol used. **c** Gating strategy used to detect CD4^+^ and CD8^+^ T AIM^+^ T cells which were defined as the addition of the frequency of cells that were either CD69^+^PD-L1^+^4-1BB^+^, CD69^+^PD-L1^+^, CD69^+^4-1BB^+^ or PD-L1^+^4-1BB^+^ after subtracting the frequency of the non-stimulated condition from the antigen stimulated conditions. Total HIV-specific AIM^+^ cells was calculated as sum of each of the four independent antigen stimulations. Total HIV-1 specific CD4^+^ (**d**) and CD8^+^ T cells (**e**) and Staphylococcal Enterotoxin B (SEB) -responsive CD4^+^ (**f**) and CD8^+^ T cells (**g**) at day 0 (baseline), 90 and 365 days after ART initiation for each randomization group (ART-control, ART + 3BNC117, ART + romidepsin (RMD) and ART + 3BNC117 + RMD) are shown. Box-and-whisker plots show median values (center line), 25th to 75th percentiles (box outline), and the range of values (whiskers). Longitudinally collected PBMCs were available and analyzed for 52 individuals from which at least one timepoint was included (*n* = 14 ART-control group, *n* = 12 in ART + 3BNC117, *n* = 13 in ART + RMD and *n* = 14 in ART + RMD + 3BNC117). *P*-values were calculated using two-sided paired Wilcoxon test. Source data are provided as a Source Data file.

In a phase 1b/2a clinical trial^17^, individuals with HIV-1 starting ART were randomized to receive: (1) ART only, (2) ART + 3BNC117 at day 7 and 21 after ART initiation, (3) ART + RMD at day 10, 17 and 24 after ART initiation or (4) ART + 3BNC117 + RMD (Fig. 1a and Supplementary Fig. 1). To determine whether bNAb treatment was associated with alterations in HIV-1-specific T cell immune responses, we applied the activation-induced marker (AIM) assay to samples obtained at baseline, 90 and 365 days after treatment initiation (Fig. 1a). Peripheral blood mononuclear cells (PBMCs) were stimulated separately with one of 4 peptide pools (Env, Gag, Nef or Pol) (Fig. 1b). Based on induced activation marker expression, HIV-1-specific CD4^+^ or CD8^+^ T cells were defined as PD-L1^+^4-1BB^+^, PD-L1^+^CD69^+^, CD69^+^4-1BB^+^ or PD-L1^+^4-1BB^+^CD69^+^ following each peptide-pool stimulation (Fig. 1c).

We found that the frequency of total HIV-1-specific CD4^+^ T cells, defined as the sum of the four HIV-1-reactive populations for each of the four antigen-stimulations, did not change significantly over time (Fig. 1d). In contrast, the frequency of total HIV-1-specific CD8^+^ T cells significantly decreased in the ART-control group from baseline to 90 (*P* = 0.0061) and 365 (*P* = 0.0020) days of ART, whereas in the three interventional groups (ART + RMD, ART + 3BNC117 and ART + 3BNC117 + RMD), we only observed non-significant decreases over 365 days of ART (Fig. 1e). By contrast, the frequency of CD4^+^ and CD8^+^ T cell responses to Staphylococcal Enterotoxin B (SEB) stimulation did not differ between groups nor over time (Fig. 1f, g). Overall, there was no difference in the total HIV-1-specific CD4^+^ T nor CD8^+^ T cells responses between the interventional groups.

**Fig. 2:**
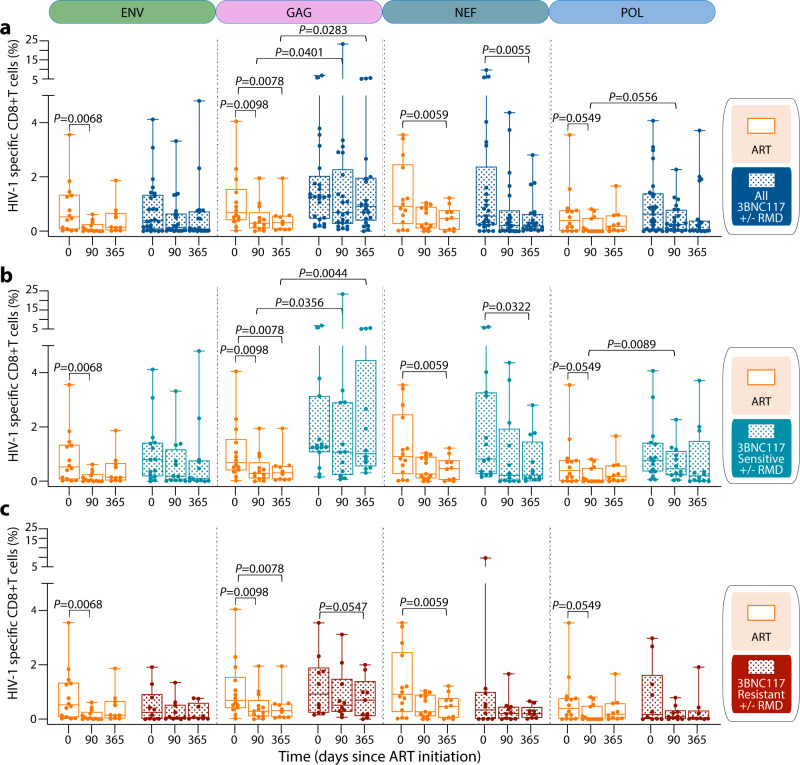
Evaluation of T HIV-specific T cell responses to four HIV-1 antigens. Net frequency of HIV-specific CD8^+^ AIM^+^ T cells for each independent stimulation (Env, Gag, Nef and Pol) plotted by box plot for **a**, ART-control and individuals receiving 3BNC117 (+/− RMD). **b** ART-control and 3BNC117 sensitive individuals receiving 3BNC117 (+/− RMD). **c** ART-control and resistant 3BNC117 individuals receiving 3BNC117 (+/− RMD). Longitudinally collected PBMCs were available and analyzed for 40 individuals from which at least one timepoint was included (*n* = 14 ART-control group, *n* = 15 3BNC117 sensitive and *n* = 11 3BNC117 resistant individuals receiving the bNAb (+/− RMD)). Box-and-whisker plots show median values (center line), 25th to 75th percentiles (box outline), and the range of values (whiskers). *P*-values were calculated using two-sided paired Wilcoxon test when comparing longitudinal data of the same group and by two-sided Mann–Whitney test when comparing two different groups. Source data are provided as a Source Data file.

Since we did not observe any detrimental effect of RMD administration on T cell immunity^17^ (and Supplementary Fig. 2a–d), and to better study the effect of the bNAb, we grouped individuals who received 3BNC117, irrespective of RMD administration. Of note, the baseline characteristics of individuals harboring pre-ART 3BNC117-sensitive viruses were comparable to those of individuals harboring pre-ART 3BNC117-resistant viruses and to the ART-control group (Supplementary Table 1). When assessing the ART-control group, we observed a decrease in the number of HIV-1-specific CD8 + T cells for all the antigens tested: Env, Gag, Nef, and Pol (Fig. 2a). In contrast, in the 3BNC117 group only Nef-specific CD8^+^ T cells declined significantly (day 365, *P* = 0.0055) (Fig. 2a). Interestingly, the frequency of Gag-specific CD8 + T cells was significantly higher in the 3BNC117 group compared to the ART-control group at 90 (*P* = 0.0401) and at 365 (*P* = 0.0283) days after ART initiation (Fig. 2a). In contrast to Gag, no difference between responses directed towards less immunodominant HIV-1 antigens was found between the ART-control and the 3BNC117 group, except for a trend towards increased Pol-specific CD8 + T cells at 90 days after ART initiation in the 3BNC117 group compared to the ART-control group (*P* = 0.0556) (Fig. 2a). These differences in HIV-1-specific CD8^+^ responses were primarily due to individuals whose plasma viruses were sensitive to 3BNC117 at baseline. 3BNC117 sensitive individuals were able to maintain significantly higher levels of Gag-specific CD8^+^ T cells compared to ART-control group at 90 (*P* = 0.0356) and 365 (*P* = 0.0044) days of ART (Fig. 2b). Notably, the sustained Gag-specific CD8^+^ T cell responses observed in 3BNC117 sensitive individuals receiving 3BNC117 could be directly attributed to the bNAb treatment, since no differences in frequencies of HIV-specific CD8^+^ T cells were observed when comparing 3BNC117 resistant individuals to the ART-control (Fig. 2c). There was also a trend towards a difference in Gag-specific CD8^+^ T cell responses between 3BNC117 sensitive and 3BNC117 resistant individuals at 365 days (*P* = 0.0804, Supplementary Fig. 2e). Of note, we did not observe significant differences in Gag-specific CD8^+^ T cells responses between individuals with recent (<6 months) vs chronic (>6 months) infection at ART initiation (Supplementary Fig. 3).

The frequency of HIV-1 specific CD4^+^ T cells was more stable over time, except for a significant decrease in Env-specific CD4^+^ T cells in the ART-control group and a decrease in Gag-specific CD4^+^ T cells in the 3BNC117 group (Supplementary Fig. 4a). However, no significant differences were observed over time between the ART-control group and individuals who were sensitive to 3BNC117 (Supplementary Fig. 4b) nor resistant to 3BNC117 (Supplementary Fig. 4c), or when comparing 3BNC117 sensitive individuals to 3BNC117 resistant individuals (Supplementary Fig. 4d).

In summary, as expected, the pool of HIV-1-specific cells within the total CD8^+^ T cell compartment contracted following ART initiation and up to 1 year of ART. Interestingly, we observed sustained HIV-1 Gag- and Pol-specific CD8^+^ T cell responses over time in individuals receiving 3BNC117 treatment at ART initiation. This immunological effect was dependent on pre-ART virus sensitivity to the bNAb.

### IFN-γ responses following Gag stimulation are associated with sensitivity to 3BNC117

**Fig. 3:**
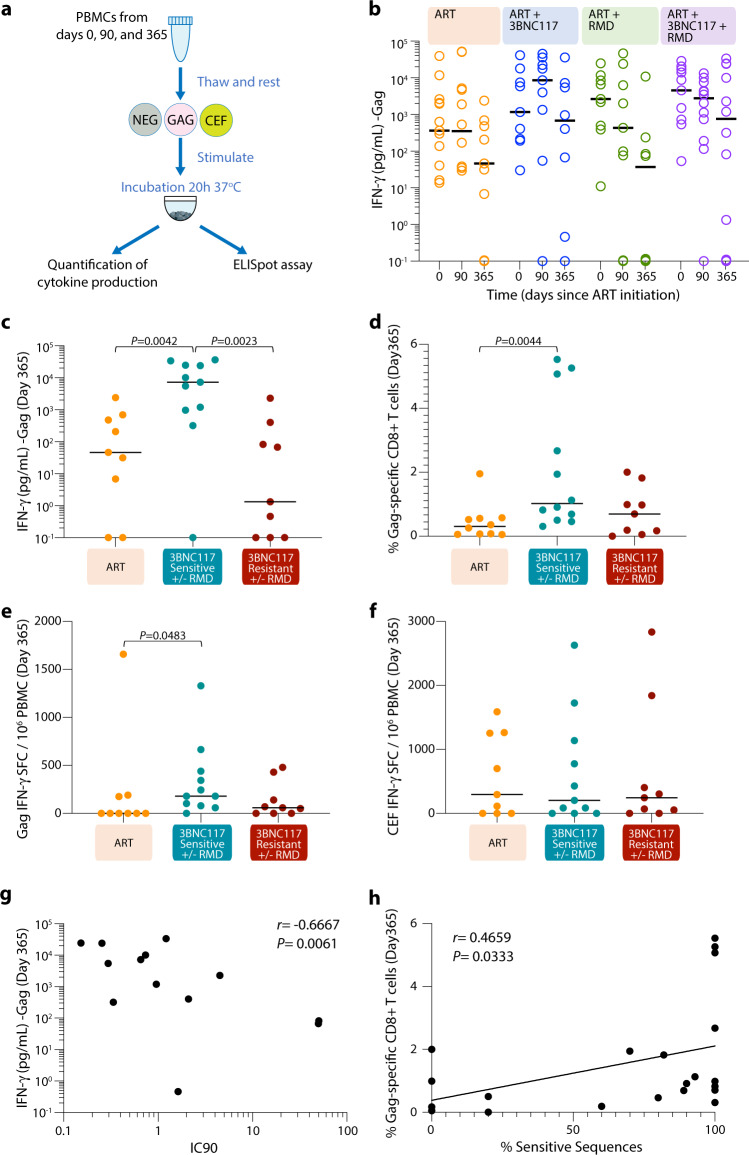
Detection of IFN-γ and correlation between T cell immunity and pre-ART sensitivity to 3BNC117. **a** Representation of the protocol followed to detect interferon-gamma (IFN-γ) release and ELISpot upon Gag stimulation. **b** Concentration of IFN-γ (pg/mL) in the supernatants from HIV-1 Gag stimulated PBMCs detected by Mesoscale for each randomization group (*n* = 13 ART-control group, *n* = 9 in ART + 3BNC117, *n* = 9 in ART + RMD and *n* = 12 in ART + RMD + 3BNC117). Concentration of IFN-γ (pg/mL) in the supernatants (*n* = 29) (**c**), frequency of specific-CD8^+^ T cells (*n* = 31) from HIV-1 Gag stimulated PBMCs (**d**) and IFN-γ ELISpot response to Gag (*n* = 29) (**e**) and to CEF (CMV, EBV and influenza virus) (*n* = 29) (**f**) at 365 days after ART initiation is shown for the following groups: ART-control group, 3BNC117 sensitive individuals receiving the bNAb and 3BNC117 resistant individuals receiving the bNAb. *P*-values were calculated using two-sided Mann–Whitney test. **g** Relationship between IFN-γ release after 365 days of ART and pre-ART 3BNC117 IC90 (µg/mL) (*n* = 16). **h** Correlation between Gag-specific CD8^+^ T cell responses after 365 days of ART and % of pre-ART sensitive sequences to 3BNC117 determined by the “bNAb-ReP” algorithm (*n* = 19). Individuals with no IFN-γ production were assigned a value of 0.1 for inclusion in the graph **b** and **c**. R and *P*-values for two-sided Spearman rank-order correlation are shown in **g** and **h**. Source data are provided as a Source Data file.

We next used supernatants from peptide-pool stimulated PBMCs in the AIM assays to detect IFN-γ release upon Gag-stimulation using a Mesoscale assay (Fig. 3a). Of note, we observed that levels of IFN-γ release correlated with the frequency of Gag-specific CD8^+^ T cells (Supplementary Fig. 5a), but not Gag-specific CD4^+^ T cells (Supplementary Fig. 5b) measured by the AIM assay. Like our AIM results, IFN-γ release following Gag stimulation tended to wane over time, especially in individuals who did not receive 3BNC117 (Fig. 3b). However, 3BNC117 sensitive individuals showed significantly higher levels of IFN-γ production after 365 days of ART compared to individuals in the ART-control group (*P* = 0.0042) as well as individuals receiving 3BCN117 whose pre-ART viruses were resistant to the bNAb (*P* = 0.0023) (Fig. 3c). The higher level of Gag-induced IFN-γ responses among 3BNC117 sensitive individuals was also present amongst the subset of trial participants that underwent analytical treatment interruption after 13 months of ART (*P* = 0.0159, Supplementary Fig. 6a). Similarly, 3BNC117 sensitive individuals showed significantly higher AIM Gag-specific CD8^+^ T cells at day 365 compared to ART-control group (*P* = 0.0044, Fig. 3d). In line with our AIM and supernatant IFN-γ release data, Gag IFN-γ SFC/10^6^ PBMC levels as determined by enzyme-linked immunosorbent spot assay (ELISPOT, Fig. 3a) after 365 days of ART, but not at baseline, was significantly higher among 3BNC117 sensitive individuals compared to ART-control group (*P* = 0.0483, Fig. 3e and Supplementary Fig. 6b), whilst CEF (CMV, EBV and influenza virus) responses were comparable at all time points between the groups (Fig. 3f and Supplementary Fig. 6c).

Thus, the observed higher IFN-γ responses among 3BNC117 sensitive individuals after 365 days of ART support the idea that bNAb administration at ART initiation maintains long-term HIV-1-specific CD8^+^ T cell immunity.

### T cell immunity correlates with 3BNC117 sensitivity

To further explore the relationship between the viruses circulating at the time of treatment initiation and the observed impact of the bNAbs on T cell responses, we measured 3BNC117-sensitivity by the PhenoSense assay and analyzed viral *env* sequences obtained by single-genome amplification.

We found a negative correlation between 3BNC117 IC90 (µg/mL) values and Pol-specific CD8^+^ T cell responses after 90 days of ART (*r* = −0.5114, *P* = 0.0301, Supplementary Fig. 7a). Moreover, IFN-γ responses upon Gag stimulation 1 year after ART initiation inversely correlated with 3BNC117 IC90 (*r* = −0.6667, *P* = 0.0061, Fig. 3g) and positively correlated with maximum percent inhibition (Supplementary Fig. 7b). Finally, the percentage of sequences sensitive to 3BNC117 observed in each participant correlated with the frequency of Gag-specific CD8^+^ T cells after 1 year of ART (*r* = 0.4659, *P* = 0.0333, Fig. 3h), as well as with the fold change of Gag-specific CD8^+^ T cells after 1 year of ART compared to baseline (Supplementary Fig. 7c). Of note, the level of the exhaustion marker PD-1 expression on CD8^+^ T cells during the administration of 3BNC117 (day 7 and day 21) was comparable between individuals with 3BNC117 sensitive and resistant- viruses (Supplementary Fig. 8). Collectively, these results suggest an association between the susceptibility of HIV-1 envelope to 3BNC117 neutralization and the level of CD8^+^ T cell immunity in individuals receiving adjunctive 3BNC117 treatment at ART initiation.

### Correlates of ART-free virologic control during treatment interruption

Out of 60 enrolled trial participants, 20 individuals underwent ATI. Their baseline characteristics are shown in Supplementary Table 2. Loss of virologic control was defined in the trial protocol as 2 consecutive plasma HIV-1 RNA measurements >5,000 copies/mL. Seven individuals maintained HIV-1 control throughout the 12-week ATI, of whom two carried a protective HLA class I allele^17^ (one in the ART-control group and one in the ART + 3BNC117 + RMD group). As reported by Gunst et al.^17^, 80% of the individuals with 3BNC117 sensitive pre-ART plasma viruses compared to 0% of individuals with 3BNC117 resistant pre-ART plasma viruses showed ART-free virological control. Considering that three individuals not receiving 3BCN117 also had virological control during ATI, we further analyzed parameters that could be associated with ART-free control during treatment interruption.

**Fig. 4:**
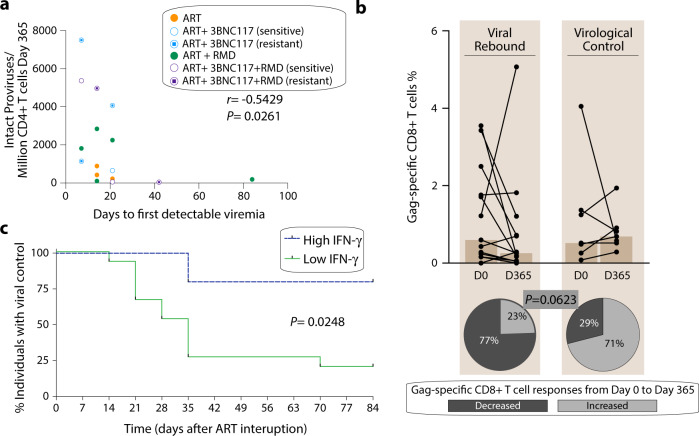
Correlation of ART-free virological control. **a** Correlation between the number of intact proviruses detected per million of CD4^+^ T cells detected after 365 days of ART (immediately preceding ATI) and days to first detectable viremia during ATI (*n* = 17). Individual dots are color-coded according to the groups. R and *P*-values were calculated using two-sided Spearman rank-order correlation. **b** Frequency of Gag-specific CD8^+^ T cell and percentage of individuals showing a decreased or increased frequency of Gag-specific CD8^+^ T cell responses (%) from Day 0 to Day 365 is shown for individuals that experienced a viral rebound (left, *n* = 13) in comparison to individuals that maintained a virological control during the ATI period (right, *n* = 7). *P*-value was calculated using two-sided Fisher’s exact test**. c** The proportion of individuals with high INF-γ (>750 pg/mL) versus low INF-γ (<750 pg/mL) who maintained viral control during 12 weeks of ATI (*n* = 20). *P*-value was calculated using log-rank test. Source data are provided as a Source Data file.

We hypothesized that the size of the HIV-1 reservoir may predict time to the first detectable viremia during ATI. In support of this, we found an inverse correlation between the number of intact proviruses determined by a modified intact proviral DNA assay (see Methods) pre-ATI and the day of first detectable viremia during ATI (*r* = −0.5429, *P* = 0.0261, Fig. 4a). Next, we investigated whether HIV-1-specific CD8^+^ T cell responses were associated with virologic control during the ATI. We observed that 5 out of the 7 (71%) individuals with virologic control compared to 3 out of 13 (23%) of individuals who experienced rebound had higher magnitude of Gag-specific CD8^+^ T cells responses after 1 year of ART (pre-ATI) compared to baseline (Fig. 4b). Of note, one of the individuals carrying a protective allele and maintaining control during the ATI, had no increase in Gag-specific CD8^+^ T cells responses after 1 year but did have increased HIV-1-specific CD8^+^ T cell responses to other HIV-1 antigens. In further support of a relationship between pre-ATI HIV-1 Gag-specific responses and the clinical outcome of the ATI, we found that high IFN-γ producers (>750 pg/mL) had superior virologic control during the ATI compared to low IFN-γ producers (<750 pg/mL) (*p* = 0.0248, Fig. 4c).

We therefore conclude that while the size of the intact HIV-1 reservoir predicted time to first detectable plasma viremia, Gag-specific CD8^+^ T cell immune responses, but not reservoir size, was associated with virological control during ART interruption.

### Long-term ART-free complete virologic control in one 3BNC117-treated individual

**Fig. 5:**
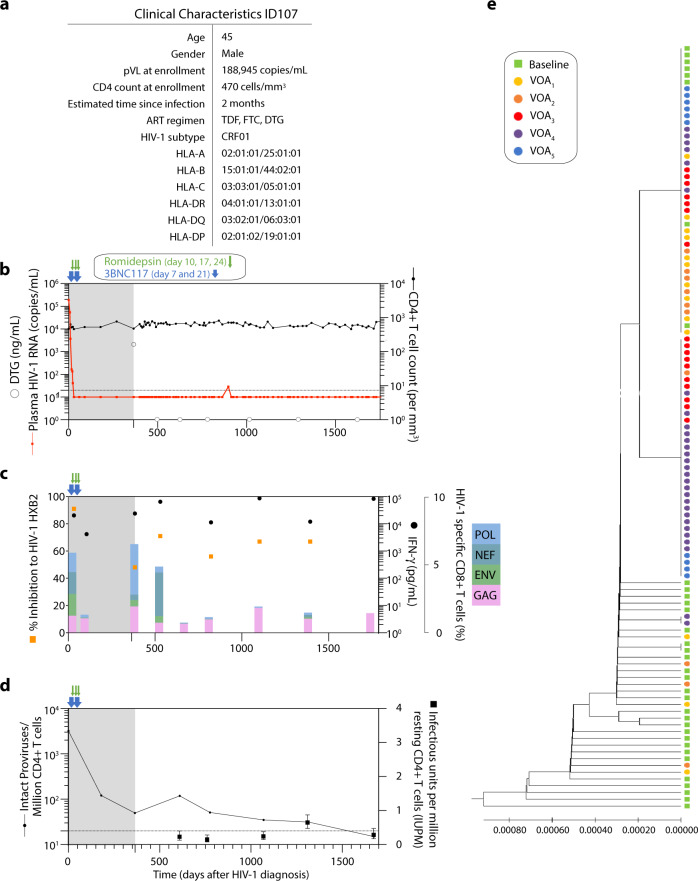
Clinical and immunological characteristics of ID107. **a** Clinical characteristics for ID107: age, gender, plasma viral load and CD4 count at enrollment, estimated time since infection, ART regimen, HIV-1 subtype and HLA typing. **b** Plasma HIV-1 RNA levels (copies/mL) and CD4+ T cell counts are shown longitudinally over the period of the interventional (gray area) and ART-free (white area) period. Time points for measurement of the concentration of dolutegravir (DTG) (ng/mL) shown as gray circles over time. **c** Frequency of HIV-1-specific CD8^+^ T cell responses to HIV-1 Env, Gag, Pol and Nef detected by the AIM assay shown as color coded in stacked bars. IFN-γ concentration following Gag stimulation shown in black dots and percentage of viral inhibition against HIV-1_HXB2_ shown in orange squares. **d** Number of intact proviruses detected per 10^6^ CD4^+^ T cells (black line) and infectious units per million resting CD4^+^ T cells (mean and 95% CI, black squares). **e** UPGMA phylogenetic tree of plasma baseline *env* sequences (green) and viral outgrowth from five different time points (VOA_1_, VOA_2_, VOA_3_, VOA_4_ and VOA_5_ at days 610, 776, 1066 1311 and 1674 after ART initiation, respectively) using Jukes-Cantor model. Source data are provided as a Source Data file.

Among the individuals who had the lowest frequency of intact proviruses per 1 million CD4^+^ T cells after 1 year of ART, ID107 also had one of the highest levels of IFN-γ production following Gag stimulation (Supplementary Fig. 9a). This individual had no plasma HIV-1 RNA measurements above 20 copies/mL during the 12-week ATI and after consultation with his treating HIV-1 physician, ID107 decided to continue off ART. Interestingly, this individual has now been able to control HIV-1 viremia, with multiple plasma samples testing negative for antiretroviral drugs, for >4 years after treatment interruption (Fig. 5a, b). ID107 received 3BNC117 in combination with RMD treatment during the clinical trial and had pre-ART plasma viruses that were sensitive to neutralization by 3BNC117. ID107 was infected with HIV-1 subtype CRF01 AE and had a plasma viral load of 188,945 copies/mL and CD4 counts of 470 cells/mm^3^ at time of enrollment (Fig. 5b). The high viral load at baseline and the absence of protective HLA alleles in this individual suggest that ID107 did not have an elite controller phenotype at study entry. We characterized the reservoir size as well as T cell immunity of ID107 over the 4 years of ART-free control. As shown in Fig. 5b plasma HIV-1 RNA remained undetectable at all time points except for a single measurement of 29 copies/mL that occurred 533 days after stopping ART. Frequencies of HIV-1 specific CD8^+^ T cells in ID107 decreased after 90 days of ART but increased again after 365 days with a more focused response towards Gag and Pol compared to the responses at baseline (Fig. 5c). During ATI, the frequency of HIV-1-specific CD8^+^T cell responses towards Pol, Nef and Env decreased, whereas a strong HIV-1 Gag specific CD8^+^ T response was maintained over time. A similar pattern was observed for Gag-induced IFN-γ production which was also sustained at very high levels throughout the treatment interruption period (Fig. 5c). The total frequency of CD4^+^ T responses was lower at baseline compared to CD8^+^ T responses, but followed a similar pattern of responses after ART initiation. Specifically, the CD4^+^ T cell responses expanded after 1 year of ART and at the beginning of ATI, with durable Gag-specific CD4^+^ T cell responses detected throughout the ATI (Supplementary Fig. 9b). Further, using autologous cells in a viral inhibition assay, we noted a strong capacity of CD8^+^ T cells to inhibit HIV-1_HXB2_ replication at enrollment (91% viral inhibition) (Fig. 5c). Although the viral inhibition capacity was lower at 1 year of ART (48% of inhibition), it increased again during the treatment interruption and was maintained over time (median of 67% inhibition, range: 56–71%) (Fig. 5c).

Finally, to characterize the latent reservoir of this individual during the treatment interruption, we performed quantitative viral outgrowth assay (qVOA) and duplex ddPCR [3dPCR] at 5 time points (Fig. 5d). The proportion of CD4^+^ T cells carrying replication-competent virus remained low around 0.2–0.3 infectious units per 1 million (IUPM) cells throughout the ATI, except for a transient 2.5-fold expansion at 1.5 years into treatment interruption which had returned to baseline levels at the following measurement 1 year later. Of note, the increase in IUPM coincided with an expansion of HIV-1-specific CD4^+^ T cells responses detected by the AIM assay (Fig. 5d and Supplementary Fig. 9b). In contrast, the intact provirus levels per million CD4^+^ T cells as assessed by 3dPCR continued to decline over time from 3216 copies/10^6^ CD4^+^ T cells at ART initiation to 50 copies pre-ATI, and to 15 copies/10^6^ CD4^+^ T cells at 3.8 years into the ATI. To better characterize the latent emerging viruses, we performed single genome amplification (SGA) *env* sequencing of viral RNA from the plasma at baseline and of viral outgrowth from 5 qVOA time points (Fig. 5e). Phylogenetic analysis of these sequences showed very low sequence diversity, with a median pairwise distance of 0.00077 at baseline as has been previously observed in early stages of HIV-1 infection. The outgrowth viruses (VOAs) showed an even lower diversity, with a median pairwise distance of 0.00039. Moreover, outgrowth viruses were closely related to pre-ART plasma viruses and virus identical to pre-ART plasma virus was found at all VOA time points. These results suggest that despite having a replication-competent reservoir, ID107 was able to maintain ART-free virologic control for >4 years of ATI.

## Discussion

The data obtained in our clinical study indicates that passive administration of 3BNC117 at the time of ART initiation enhanced HIV-1-specific cellular immunity. The mechanism underlying this observation may be due to a vaccinal effect which is believed to involve formation of antibody:HIV-1-antigen immune complexes and their activation of antigen-presenting cells resulting in enhanced presentation of HIV-1 antigens to T cells. The increased HIV-1-specific CD8^+^ T cell responses persisted for up to 1 year after 3BNC117 administration and was associated with ART-free control during a treatment interruption.

HIV-1-specific cytotoxic CD8^+^ T cells responses play a crucial role in the initial decay of viremia upon infection and have been strongly associated with control of HIV-1 infection^18,19^. Therefore, it is believed that the induction of CD8^+^ T cell activity would prompt the clearance of infected cells. Recently, the administration of bNAbs has been associated with enhanced virus-specific T cell immunity in infected non-human primates^15,20^. Recent work in mouse models revealed that CD8^+^ T cell responses can be induced by exogenous antigen processing via non-cross presentation^21^. Additionally, increased T cell immunity specific for HIV-1 was observed in a clinical trial where individuals received bNAb therapy during an ATI^14^. Our data indicate that individuals harboring antibody-sensitive and receiving the antibody 3BNC117 at ART initiation maintained HIV-1-specific T cell immunity for up to 1 year. Specifically, we observed sustained HIV-1 Gag-specific CD8^+^ T cell responses which were consistently observed across three different immunological assays. In line with our results in the ART-control group, T cell responses to HIV-1 infection have been shown to decay rapidly in the months after ART initiation^2,22,23^. PD-1 expression on CD8^+^ T cells after the administration of 3BNC117 (day 7 and day 21) was comparable between individuals with 3BNC117 sensitive and 3BNC117 resistant- viruses as well as ART controls, suggesting that the bNAb-mediated effect on HIV-1-specific CD8^+^ T cells responses was not due to less antigen-driven stimulation and limited exhaustion of CD8^+^ T cells in the 3BNC117 sensitive group. Rather, the sustained HIV-1 Gag-specific CD8^+^ T cell responses observed in 3BNC117 sensitive individuals may be the direct result of 3BNC117 treatment. This is also supported by the responses to control antigen-stimulations with SEB and CEF which did not differ between the groups nor over time, strongly suggesting that the immunological effect of 3BNC117 is HIV-1-specific and not due an untargeted immunological mechanism. Additionally, in our study 3BNC117 was administered at ART initiation, when levels of plasma viremia and frequencies of infected cells are high, maximizing the degree of antigen exposure and the potential for antibody-mediated immune enhancement.

The more sustained HIV-1 specific CD8^+^ T cell responses after bNAb administration, were predominantly targeted towards HIV-1 Gag − the dominant protein contained in the viral particle^24^. Interestingly, CD8^+^ T cell responses are primarily targeted towards HIV-1 Gag in elite controllers, whereas CD8^+^ T cell responses biased to HIV-1 Env are associated with poor virologic control^25–27^. Thus, there is a biological rationale for the dominant Gag-specific CD8^+^ T cell response observed among 3BNC117-treated individuals. In future studies, it would be of great interest to study changes in HIV-1- specific responses in lymphoid tissues to gain a deeper insight into the mechanisms controlling viral replication in the absence of ART.

Our study also details the characteristics of an individual with 3BNC117-sensitive virus who received early bNAb treatment along with ART and RMD and who has maintained complete ART-free virologic control for over 4 years ongoing. We cannot definitely determine whether the ART-free HIV-1 control observed for ID107 was due to the early intervention or not, but what is unique about this post-treatment controller is that we have followed the individual from the time of primary HIV-1 infection, through ART initiation and viral suppression, and now during ART interruption. We have screened for the presence of antiretroviral drugs in plasma multiple times during follow-up without finding any trace of antivirals. Replication-competent virus has been isolated from PBMCs collected from this individual at five different time points during the ART-free period. However, based on *env* sequencing, the viral reservoir does not evolve over time and the frequency of intact proviruses is steadily declining. The CD8^+^ T cell responses are increasingly targeting HIV-1 Gag versus targeting HIV-1 Env, Pol, or Nef, and the magnitude of the HIV-1 Gag appears to have stabilized at a low level around 1%. How this targeting pattern has impacted the genomic location and integration of the remaining intact proviruses will be explored in future studies.

While in vitro genotypic or phenotypic sensitivity testing for CD4-binding-site bNAbs such as 3BNC117 has been reported to have some limitations in accurately predicting in vivo sensitivity^28^, in vitro sensitivity of plasma viruses to bNAb-mediated neutralization among ART naïve individuals accurately predicts the antiviral activity of bNAbs in vivo^29–31^. Consistent with the published literature, our comparative findings show that in vitro phenotypic and in silico genotypic sensitivity testing on pre-ART plasma accurately identifies individuals who respond (sensitive) or do not respond (resistant) virologically and immunologically to 3BNC117 administration in vivo.

In conclusion, we demonstrated maintained CD8^+^ cell responses in people with HIV-1 who received 3BNC117 infusions at the time of ART initiation. Notably, the sustained CD8^+^ T cell responses were dependent upon pre-ART plasma virus sensitivity to neutralization by 3BNC117. We further found that Gag-induced IFN-γ release prior to the ATI predicted ART-free virologic control and may represent a potential biomarker for post-treatment control in future studies. Finally, we detail the intriguing case of a 3BNC117-treated individual who has maintained ART-free virologic control for >4 years ongoing. We conclude that early administration of potent bNAbs in individuals starting ART may, due to sustained cellular immunity, be an essential component in strategies aiming to induce long-term ART-free virologic control. In future studies, intensive sampling from blood and tissue could help unravel the mechanisms behind the maintenance of CD8^+^ T cell following bNAb administration at ART initiation.

## Methods

### Study design and samples

In a phase Ib/IIa multicenter, randomized controlled trial (the eCLEAR study), newly HIV-1 diagnosed adults (5 females and 50 males) aged 18–65 years were randomized into one of four groups: (a) ART-control, (b) ART + 3BNC117 (30 mg/kg) at day 7 and 21 after ART initiation, (c) ART + RMD (5 mg/m2) at day 10, 17 and 24 after ART initiation, or (d) ART + 3BNC117 + RMD^1^. Participants were followed for 365 days with an optional 12-week analytical treatment interruption (ATI) starting at day 400. Recent HIV-1 infection was defined as <6 months from time of infection and “chronic” infection as > = 6 months from time of infection. Before any study-related procedures, written informed consent was obtained from the participant. The study was conducted in accordance with Good Clinical Practice and reported in accordance with the CONSORT 2010 statement^32^. The protocol was approved by the Danish Medicine Authorities in Denmark and the Medicines (#2016053184) and Healthcare Products Regulatory Agency (MHRA) in the United Kingdom (#31883/0001/001-0001), and the National Committee on Health Research Ethics in Denmark (#1-10-72-110-16) and National Health Authority in the United Kingdom (#241439). Study data were collected and managed in Research Electronic Data Capture (REDCap) electronic data capture tools hosted at the Clinical Trial Unit, Department of Clinical Medicine, Aarhus University, Aarhus, Denmark^33,34^. The study was monitored by the Danish Good Clinical Practice Units (https://gcp-enhed.dk/english/) from screening to final visit. Participants were reimbursed for transport expenses relating to the study, but otherwise did not receive any financial compensation for participating in the study except from compensation for lost earnings during study visits.

### Activation-induced cell marker (AIM) assay

Cryopreserved PBMCs were thawed, washed and rested at 37 °C for 3 h. Cells were then plated into wells of a 96-well plate, at a total of 1 × 10^6^ cells per well. For each assay, six conditions were used: no exogenous stimulation with 0.32% of Dimethyl sulfoxide (DMSO) as negative control, four HIV-1 antigen stimulations and staphylococcal enteroxin B (SEB, 1 μg/mL) as positive control. The antigen stimulations were overlapping peptide pools corresponding to HIV-Gag (JPT, PM-HIV-Gag), HIV-ENV (JPT, PM-HIV-ENV), HIV-NEF (JPT, PM-HIV-Nef) and HIV-POL (JPT, PM-HIV-Pol) used at a final concentration of 2 μg/mL of total peptide. Following 20 h incubation at 37 °C, supernatants were harvested for cytokine quantification and cells were washed with PBS and stained for viability with Near IR Live Dead (Invitogen, L10119) for 20 min. Cells were then incubated with Human TruStain FcX (BioLegend) in PBS 2% FBS for 10 min and stained 30 min with surface markers antibodies: CD3 (1 μL PerCP/Cy5.5 anti-human CD3, SK7, BioLegend), CD4 (2 μL, BV650 anti-human CD4, RPA-T4, BioLegend), CD8 (1 μL, BV605 anti-human CD8a, RPA-T8, BioLegend), 4-1BB (2.5 μL, PE anti-human CD137, BioLegend) CD69 (1 μL, APC anti-human CD69, FN50, BioLegend) and PD-L1 (2.5 μL, BV421 anti-human CD274, B7-H1, BioLegend). Cells were washed twice and acquired on an MACS Quant Analyzer 16. Data were analyzed using FlowJo 10.7.2. The frequency of antigen-specific cells (AIM^+^ cells) was determined by subtracting the frequency of the non-stimulation condition (NEG), which was similarly distributed between groups (Supplementary Fig. 10), from each antigen stimulated condition (ENV, GAG, NEF or POL). AIM^+^ cells were defined as the frequency of cells that were either CD69^+^PD-L1^+^4-1BB^+^, CD69^+^PD-L1^+^, CD69^+^4-1BB^+^ or PD-L1^+^4-1BB^+^. An example of how the frequency of Gag-specific CD4^+^ and CD8^+^ AIM^+^ T cells was calculated is presented in Supplementary Table 3. Total HIV-specific AIM^+^ cells was calculated as summation of each of the four independent antigen stimulations (ENV + GAG + NEF + POL).

### Cytokine detection

IFN-γ was measured in supernatants from the AIM assay using Meso Scale Discovery (MSD) V-PLEX Plus Proinflammatory Panel 1 (catalog number K15049G-1) according to the manufacturer’s instructions.

### IFN-γ enzyme-linked immunosorbent spot assay (ELISPOT)

Cryopreserved PBMCs were thawed, washed and rested at 37 °C for 3–5 h before plating 100,000 live cells per well in IFN-γ ELISPOT 96-well polyvinylidene plates (Millipore). PBMC were stimulated with were overlapping peptide pools corresponding to HIV-Gag (JPT, PM-HIV-Gag) and CEF (CMV, EBV and influenza virus) (JPT, PM-CEF-S-3) used at a final concentration of 2 μg/mL of total peptide in duplicates. The IFN-γ Mabtech kit was used, according to the manufacturer’s instructions. Spots were counted using an automated ELISPOT reader system (Immuno- Spot S6 Versa; CTL, Germany), and the magnitude of responses was expressed as spot-forming cells (SFC) per million input cells. The threshold for positive responses was defined as at least five spots per well, responses exceeding the “mean number of spots in negative-control wells plus three standard deviations of the negative-control wells” and “three times the mean of negative-control wells,” whichever was higher.

### 3BNC117 sensitivity

3BNC117 sensitivity was determined using the LabCorp Monogram Biosciences PhenoSense® HIV Monoclonal Antibody Assay on pre-ART plasma RNA viruses. Viruses were defined as sensitive to 3BNC117 when IC90 < 1.5 ug/mL and the maximum percent inhibition (MPI) >98%. When the PhenoSense assay did not yield a result due to lack of amplification, *env* sequences obtained by single genome amplification (SGA) of plasma RNA viruses from baseline were analyzed for the presence of mutations predicted to confer 3BNC117 resistance as previously described^17^.

Another genotypic prediction algorithm, the bNAb ReP^35^ was applied on SGA envelope sequences from all participants, the outcome of this algorithm is a probability of sensitivity for each sequence. We defined a sequence as being sensitive when the probability of sensitivity was predicted to >80% and a participant was defined as harboring sensitive virus when >90% of the sequences were sensitive. A least 26 sequences were analyzed for each participant.

### HLA typing

Human leukocyte antigen (HLA) class I (HLA-A, -B, -C) and II (HLA-DR, -DQ, -DP) alleles were genotyped at the American Safety and Health Institute-accredited laboratory HistoGenetics (Ossining, New York, US) using sequence-based typing.

### 3dPCR

HIV-1 reservoir size was assessed 365 days after ART initiation using a duplexed droplet digital PCR (3dPCR) based on the Intact Proviral DNA Assay (IPDA)^36^ as previously described^1^. Briefly, two primer/probe sets, located in the Packaging Signal (Ψ) and the Rev Response Element (RRE) in *envelope* used to selectively quantify presumed intact (double-positive) proviruses. The primer/probe set targeting Ψ was as described in the IPDA^36^ while primer/probe set in the RRE represented a secondary location for this assay^37^ that is located slightly downstream of the original IPDA location. Where necessary we adapted primer/probe sequences to accommodate HIV polymorphism^17^.

### In vitro viral inhibition assay

CD8^+^ T-cell mediated viral inhibition capacity was measured at 1:1 CD8^+^ effector to CD4^+^ target ratio. Cryopreserved PBMCs obtained after 12 months into ART were used to obtained CD8^+^ cells by magnetic bead separation (Cat #130-045-201 Milteny Biotec) according to the manufacturer’s protocol. CD8^+^-depleted cells (CD4^+^-enriched fraction) were stimulated with Phytohemagglutinin (PHA, Thermo Scientific™ Remel™ PHA Purified #R30852801) (5 µg/mL) in RPMI glutamine; 1% streptomycin and penicillin; 10% fetal calf serum. After 3 days of stimulation, the CD4-enriched fraction was infected by spinoculation with HIV-1_HXB2_ laboratory-adapted strain and autologous HIV-1 viruses a multiplicity of infection (MOI) of 0.01 as reported previously^38,39^. Non-infected CD4^+^ T cells were included as negative controls and infected CD4^+^ T cells without CD8^+^ T cells were included as “100% infected” controls. After infection, CD4^+^ T cells were cocultured with unstimulated CD8^+^ T cells obtained from cryopreserved PBMCs at ART initiation, 12 months into ART and during the treatment interruption (at days 610, 776, 1066 and 1311 after ART initiation) by positive magnetic bead separation. The culture medium for this assay was RPMI media with L-glutamine; 1% streptomycin and penicillin; 10% fetal calf serum; recombinant human IL-2 (50 U/mL). Cultures were performed in triplicates at ratio 1:1 (CD8:CD4) and they were harvested after 7 days. Viral replication was measured as the production of HIV-1 antigen p24 in culture supernatants (pg p24/mL) by ELISA as previously described^40,41^, and viral inhibition was expressed as a percentage with respect to the positive control.

### Viral outgrowth assay

Viral outgrowth assays were performed as previously described^42–45^ with the following modifications. Total CD4^+^ T cells were enriched from 550 million cryopreserved PBMCs by negative depletion using Miltenyi CD4^+^ T Cell Isolation Kit (Cat #130-096-533) according to the manufacturer’s protocol. All cell incubations were performed at 37 °C unless otherwise noted. The culture medium for this assay was RPMI with L-glutamine; 1% streptomycin and penicillin; 10% fetal calf serum; recombinant human IL-2 (100 U/mL) (Gibco #PHC0027); conditioned media from a mix lymphocyte reaction culture as described in^46,47^.

On day 0 CD4^+^ T cells were seeded at 50,000 cells/well in round-bottom 96-well plates and stimulated with irradiated allogeneic PBMCs from HIV-negative healthy donors and phytohemagglutinin (1 μg/mL) (Thermo Scientific™ Remel™ PHA Purified #R30852801 2 mg). After 48 h, the cells were extensively washed to remove PHA and 10,000 MOLT-4/CCR5 cells (were added to each well). On days 5, 7 and 9, 75% of the culture media per well was replenished with fresh media with an additional 10,000 MOLT-4/CCR5 cells were added to each well with the fresh media on day 9. On day 12, the cell supernatant from each well was harvested and the number of wells containing replication competent HIV was assessed by incubation of the supernatant with TZM-bl cells via firefly luciferase reporter gene activity^46^. On day 15, wells positive for luciferase activity was determined using the Britelite™ plus Reporter Gene Assay System, 100 mL (Perkin Elmer #6066761). Estimated frequencies of cells with replication-competent HIV were calculated using limiting dilution analysis as previously described^47^. MOLT-4/CCR5 (Cat No 4984) and TZM-bl cells (Cat No. 8129) were obtained from the NIH AIDS Reagent Program.

### Sequence analysis for ID107

For ID107 a sequence analysis including baseline plasma virus and outgrowth virus was conducted. The baseline plasma sequences used for 3BNC117 sensitivity testing was also used for this analysis (described above). For outgrowth virus, viral RNA was extracted from supernatant from luciferase positive QVOA wells by using QIAamp Viral RNA mini kit (Qiagen). cDNA synthesis, bulk nested PCR with gene-specific primers, library preparation and sequencing were performed as previously described^17^. Maximum likelihood phylogenetic tree and pairwise distance was generated using MEGA^48^.

### Statistics and reproducibility

Two-sided Wilcoxon matched paired test and two-sided Mann–Whitney test were used to analyze non-parametric outcomes within-and between groups, respectively. Fisher’s exact test was used to analyze contingency tables. Data are presented as median with range as indicated in each respective figure legend. The log-rank test was used to compare the proportion of individuals with high INF-γ versus low INF-γ that maintained viral control during 12 weeks of ATI. For correlations, two-sided Spearman’s correlation coefficient was used. *P*-values < 0.05 were considered significant. We used GraphPad Prism version 8.4.3 for Mac (San Diego, CA) for statistical analyses.

We used the full analysis set, comprising all individuals receiving at least one dose of active treatment with assessable data. No data were excluded from the analyses, the *n* size is indicated in each respective figure legend since the sample availability was different depending on the assay. The analyses performed on primary and secondary endpoints were prespecified in the protocol, and no exploratory analyses were done, hence no corrections for multiple comparisons were made. The results were not disaggregated by sex.

### Reporting summary

Further information on research design is available in the Nature Research Reporting Summary linked to this article.

## Supplementary information

Supplementary Information

Reporting summary

## Data Availability

Clinical data are not available for download due to privacy/ethical restrictions under the EU GDPR. Specific requests for access to the trial data may be sent to olesoega@rm.dk and access may be provided to a named individual in agreement with the rules and regulations of the Danish Data Protection agency and the National Committee on Health Research Ethics. The experimental data generated in this study in the Source Data file. The sequences generated in this study have been deposited in the GenBank database under accession codes OP031483-OP031595. Source data are provided with this paper.

## Source data

Source Data
