# Supplementary material for: Administration of broadly neutralizing anti-HIV-1 antibodies at ART initiation maintains long-term CD8+ T cell immunity
Source: Nat Commun. 2022 Oct 29;13:6473. doi: 10.1038/s41467-022-34171-2 (PMC9617872; doi:10.1038/s41467-022-34171-2)
Supplement: Supplementary file 1 — Supplementary Information [file 41467_2022_34171_MOESM1_ESM.pdf]

Administration of broadly neutralizing anti-HIV-1 antibodies at ART initiation maintains long-term CD8+ T cell immunity

Supplementary Figure 1

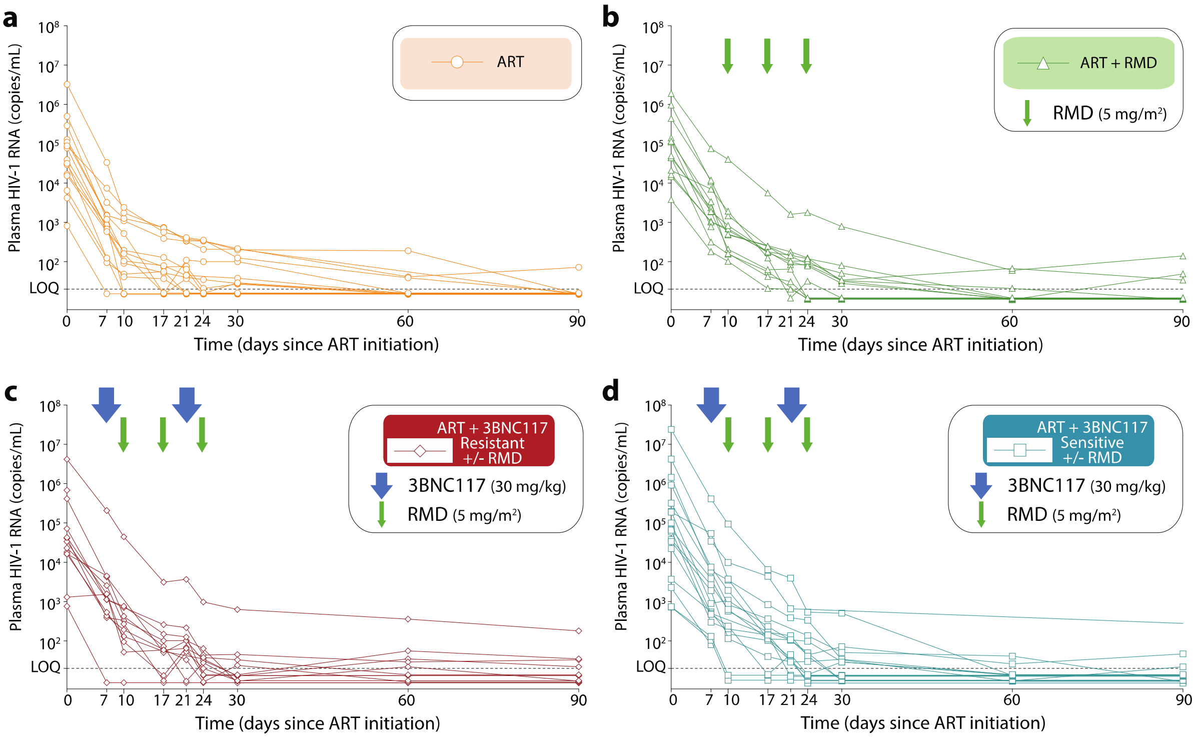

Supplementary Fig. 1: Decay of plasma HIV-1 RNA levels following ART initiation. Individual plasma HIV-1 RNA levels following ART initiation is shown for the ART-control group (a), ART+RMD group (b), 3BNC117 resistant individuals receiving 3BNC117 (+/-RMD) (c) and 3BNC117 sensitive individuals receiving 3BNC117 (+/-RMD) (d). The arrows represent 3BNC117 (blue) and RMD (green) infusion time points.

Supplementary Figure 2

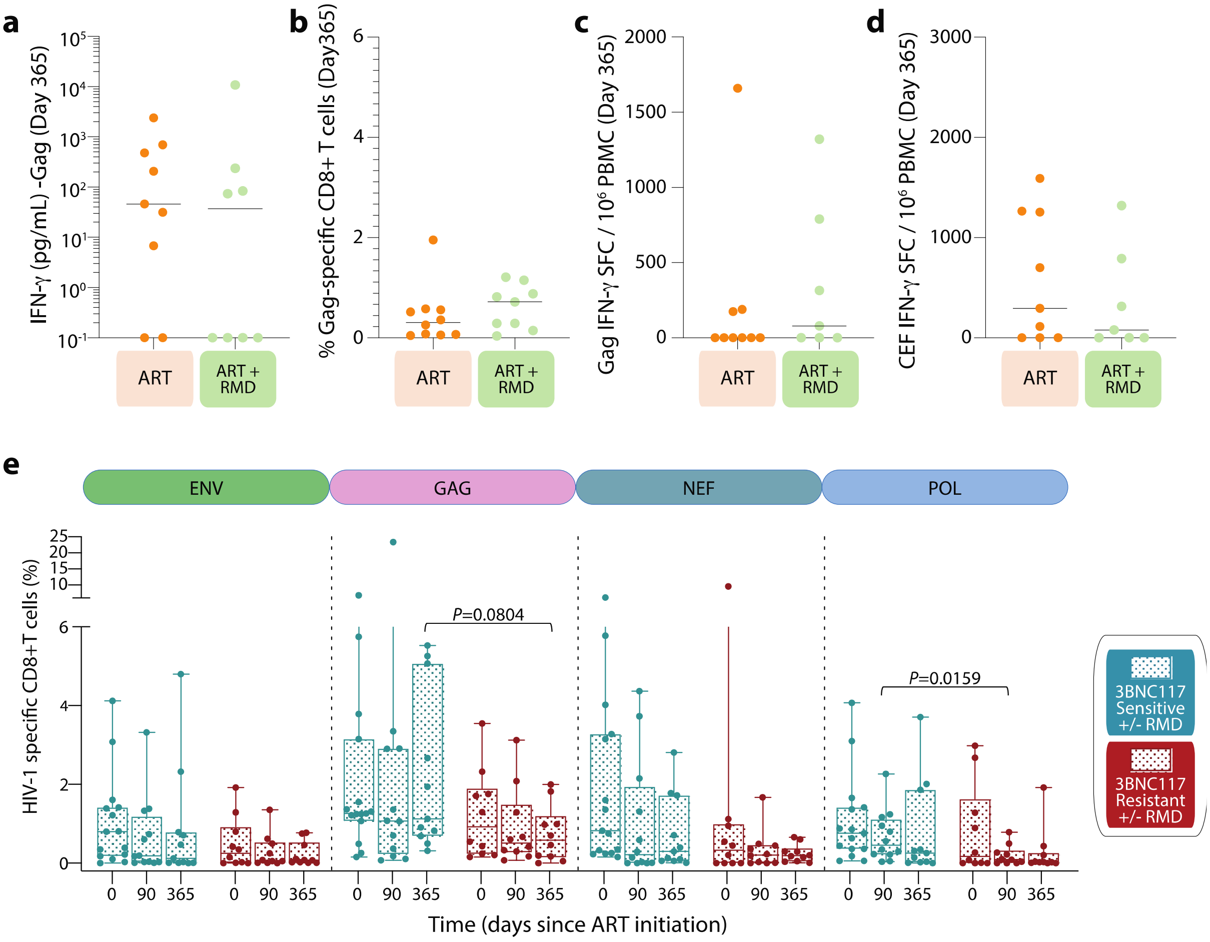

Supplementary Fig. 2: Detected T cell responses in ART and ART+RMD groups and comparison between HIV-specific T cell responses in 3BNC117 sensitive and 3BNC117 resistant individuals receiving 3BNC117. Concentration of IFN-γ (pg/mL) in the supernatants (**a**), frequency of specific-CD8$^+$ T cells (**b**) from Gag stimulated PBMCs and IFN-γ ELISpot response to Gag (**c**) and to CEF (**d**) at 365 days after ART initiation is shown for the ART-control group and ART+RMD. **e,** Frequency of HIV-specific CD8$^+$ AIM$^+$ T cells for each independent stimulation (Env, Gag, Nef and Pol) is shown for 3BNC117 sensitive and 3BNC117 resistant individuals receiving 3BNC117 (+/- RMD). Box-and-whisker plots show median values (center line), 25th to 75th percentiles (box outline), and the range of values (whiskers). P values were calculated using two-sided Mann-Whitney test. Longitudinally collected PBMCs were available and analyzed for 26 individuals from which at least one timepoint was included (n=15 3BNC117 sensitive and n=11 3BNC117 resistant individuals receiving the bNAb (+/- RMD)). P values were calculated by Mann Whitney test when comparing two different groups.

Supplementary Figure 3

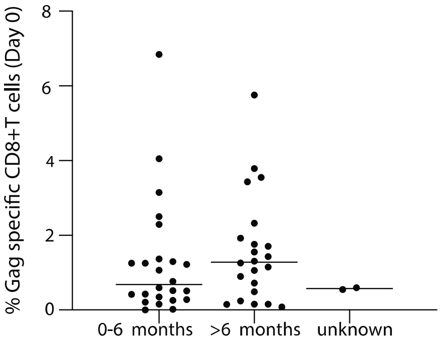

Supplementary Fig. 3: Frequency of AIM Gag-specific CD8$^+$ T cell at ART-initiation by time of infection. The frequency of Gag-specific CD8$^+$ AIM$^+$ T cells is shown for individuals diagnosed with recent (<6months), chronic (>6 months) infection or unknown time since infection.

Supplementary Figure 4

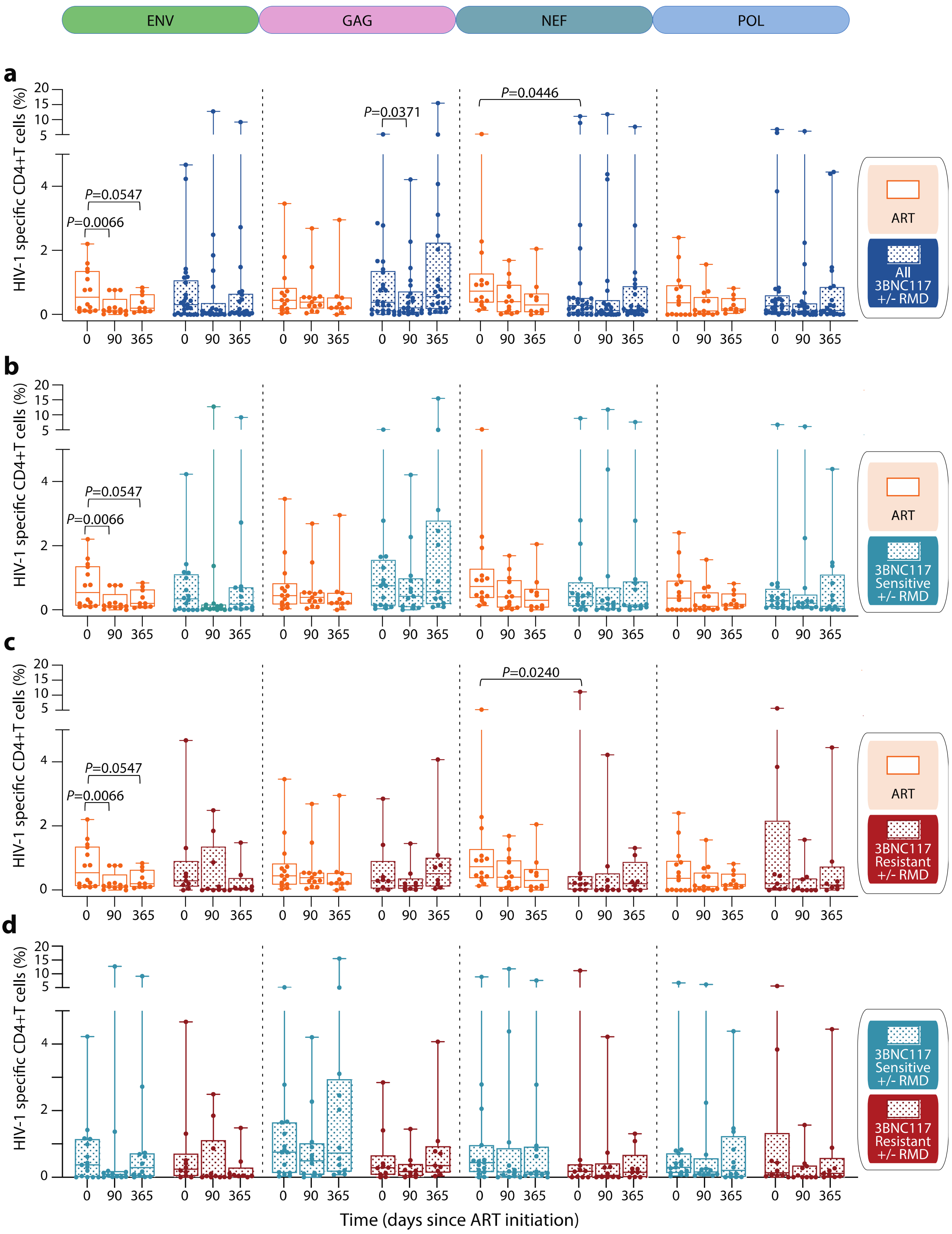

Supplementary Fig. 4: Evaluation of CD4$^+$ T HIV-specific T cell responses to four HIV-1 antigens. Net frequency of HIV-specific CD4$^+$ AIM$^+$ T cells for each independent stimulation (Env, Gag, Nef and Pol) plotted by box plot for **a,** ART-control and individuals receiving 3BNC117 (+/- RMD). **b,** ART-control and 3BNC117 sensitive individuals receiving 3BNC117 (+/- RMD). **c,** ART-control and 3BNC117 resistant individuals receiving 3BNC117 (+/- RMD).

d, 3BNC117 sensitive individuals receiving 3BNC117 (+/- RMD) (and 3BNC117 resistant individuals receiving 3BNC117 (+/- RMD). Longitudinally collected PBMCs were available and analyzed for 40 individuals from which at least one timepoint was included (n=14 ART-control group, n= 15 3BNC117 sensitive and n=11 3BNC117 resistant individuals receiving the bNAb (+/- RMD)). Box-and-whisker plots show median values (center line), 25th to 75th percentiles (box outline), and the range of values (whiskers). P values were calculated using two-sided paired Wilcoxon test when comparing longitudinal data of the same group and by two-sided Mann-Whitney test when comparing two different groups.

Supplementary Figure 5

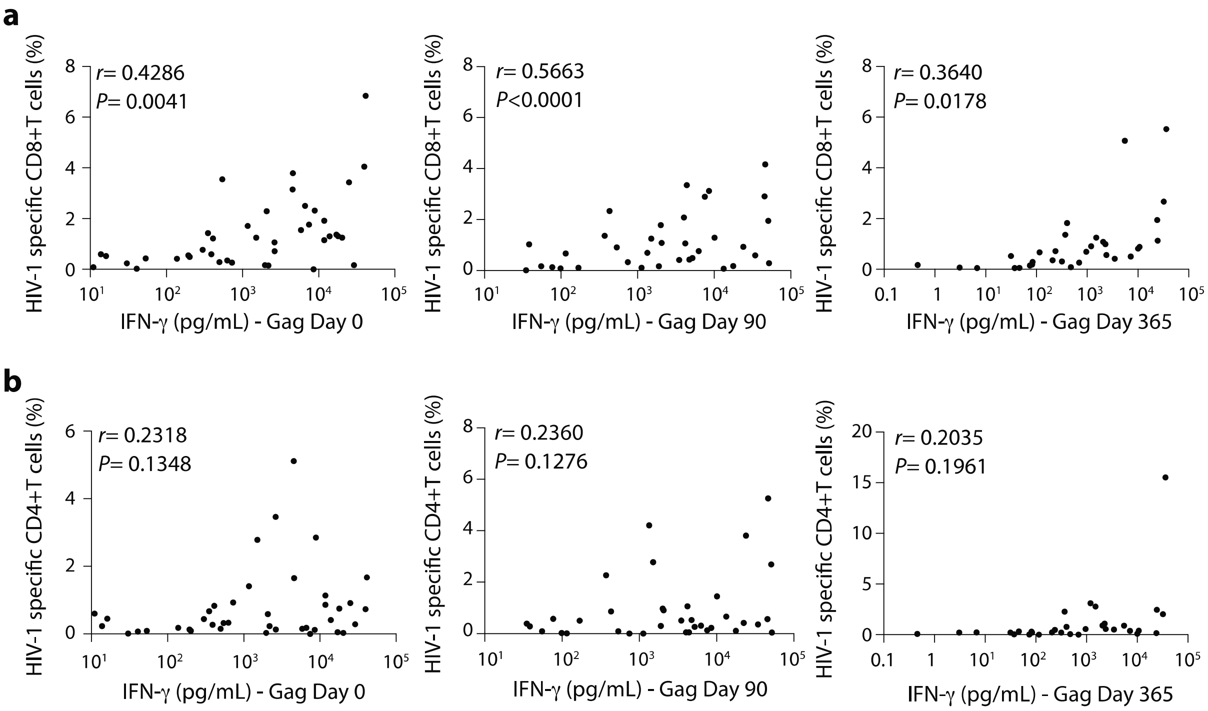

Supplementary Fig. 5: Correlation between AIM assay and IFN-γ release. Relationship between the frequency (%) of Gag-specific **a,** CD8$^+$ T and **b**, CD4+ T cells and concentration of IFN-γ (pg/mL) detected at, day 0 (baseline) (left), day 90 (middle) and, at day 365 (right) after ART initiation. R and P values for two-sided Spearman rank-order correlation are shown.

Supplementary Figure 6

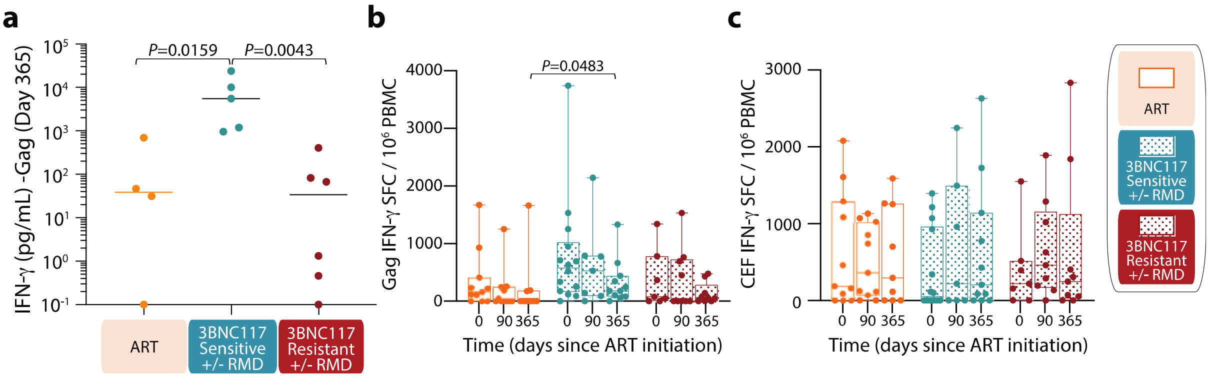

Supplementary Fig. 6: IFN-γ release in individuals undergoing ATI. a, Concentration of IFN-γ (pg/mL) in the supernatants from HIV-1 Gag stimulated PBMCs at day 365 is shown for individuals that underwent analytical treatment interruption (n=4 ART-control group, n=4 3BNC117 sensitive individuals receiving 3BNC117 and n=6 3BNC117 resistant individuals receiving 3BNC117). Longitudinal IFN-γ ELISpot response to Gag (**b**) and to CEF (**c**) is shown for the following groups: ART-control group, 3BNC117 sensitive individuals and 3BNC117 resistant individuals receiving the bNAb. Box-and-whisker plots show median values (center line), 25th to 75th percentiles (box outline), and the range of values (whiskers). P values were calculate using two-sided Mann-Whitney test. Longitudinally collected PBMCs were available and analyzed for 34 individuals from which at least one timepoint was included: n=11 for ART-control group, n= 14 for 3BNC117 sensitive the bNAb and n=9 for 3BNC117 resistant receiving the bNAb.

Supplementary Figure 7

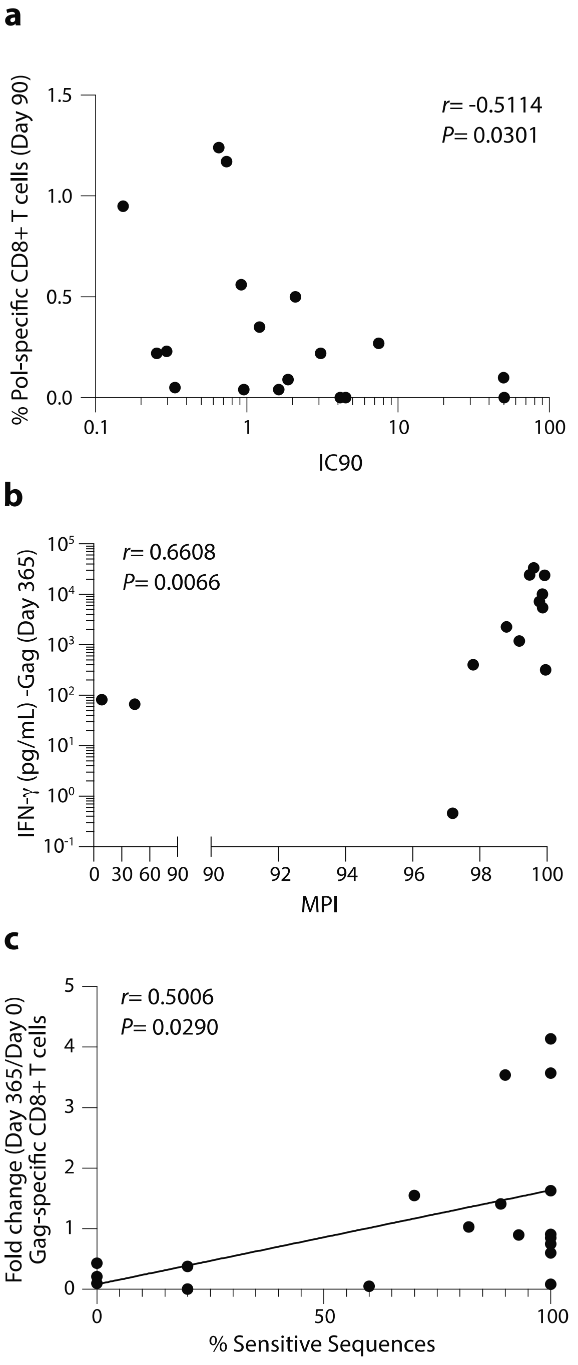

Supplementary Fig. 7: Correlation between T cell immunity and pre-ART sensitivity to 3BNC117. a, Relationship between Pol-specific $CD8^+$ T cells at day 90 and pre-ART 3BNC117 IC90 (µg/mL) from individuals in ART + 3BNC117 +/- RMD (n=18)**. b,** Relationship between IFN-γ concentration 365 days of ART and pre-ART 3BNC117 maximum percent inhibition (MPI) (n=17). **c,** Correlation between fold change of Gag-specific $CD8^+$ T cell responses 365 days of ART compared to % of pre-ART sensitive sequences to 3BNC117 determined by the "bNAb-ReP" algorithm (n=19). R and P values for two-sided Spearman rank-order correlation are shown.

Supplementary Figure 8

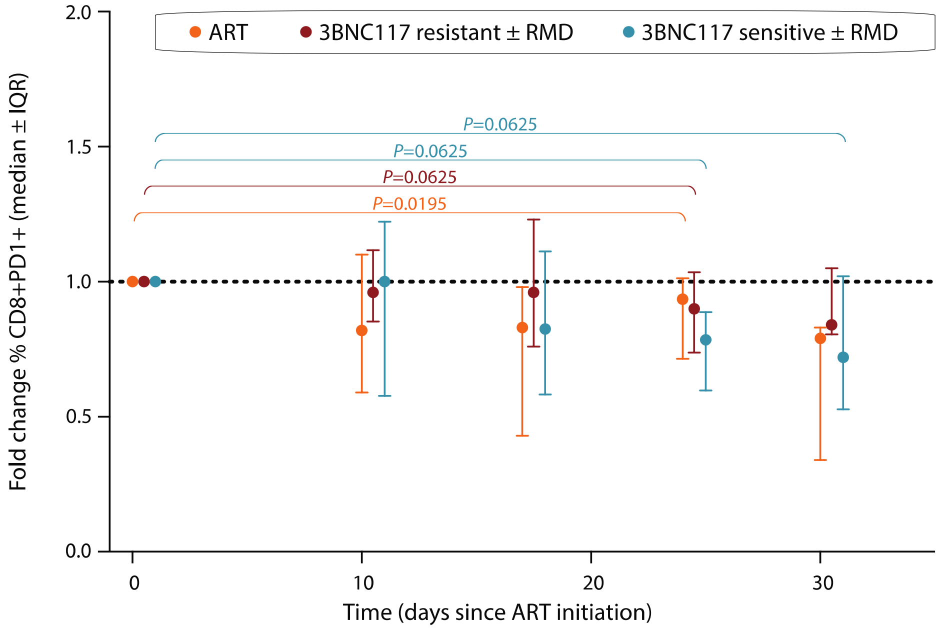

Supplementary Fig. 8: Fold change in PD-1 expressing CD8$^+$ T cells during the administration of 3BNC117. The fold change in level of CD8$^+$ T cells expressing the exhaustion marker PD-1 during the administration of 3BNC117 (day 7 and day 21) is shown for ART-control group (n=7) 3BNC117 sensitive (n=10) and 3BNC117 resistant- (n=9) individuals receiving 3BNC117. Data are presented as median values and IQR. P values comparing longitudinal data of the same group were calculated using two-sided paired Wilcoxon test.

Supplementary Figure 9

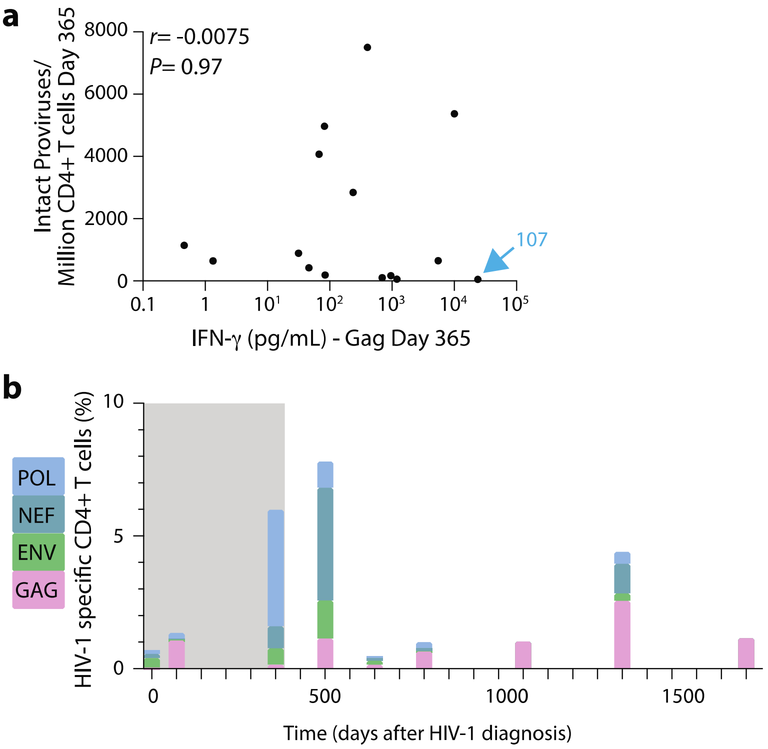

Supplementary Fig. 9: Further characterization of ID107. a, Representation of the number of intact proviruses detected per 10^6 $CD4^+$ T cells and IFN-γ production 365 days of ART. *P* values calculated using two-sided Spearman's correlation coefficient. **b**, Frequency of HIV-1-specific $CD4^+$ T cell responses to HIV-1 Env, Gag, Pol and Nef detected by the AIM assay shown color coded in stacked bars during the interventional (grey area) and ART-free (white area) period.

Supplementary Figure 10

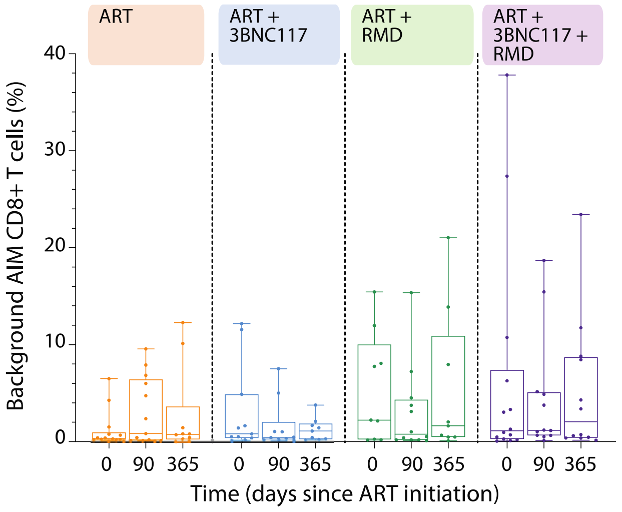

Supplementary Fig. 10: Background frequency of non-specific reactive CD8$^+$ T cells. Frequency of background AIM$^+$ calculated as the sum of the frequency of the negative well for the populations considered as antigen-specific cells (CD69$^+$PD-L1$^+$4-1BB$^+$, CD69$^+$PD-L1$^+$, CD69$^+$4-1BB$^+$ or PD-L1$^+$4-1BB$^+$) is shown for ART-control group (n=14), ART + 3BNC117 (n= 12), in ART + RMD (n=13) and ART + RMD + 3BNC117 (n=14). Box-and-whisker plots show median values (center line), 25th to 75th percentiles (box outline), and the range of values (whiskers).

Supplementary Table 1. Baseline characteristics for the ART, RMD, 3BNC117 sensitive and resistant groups

	ART (n=15)	ART +RMD (n=13)	ART +3BNC117+/-RMD sensitive (n=18)	ART +3BNC117+/-RMD resistant (n=13)
Age - yr	33 (25-58)	32 (18-68)	43 (22-58)	37 (25-69)
Female sex	04 (27)	01 (08)	00 (00)	00 (00)
Race				
Asian	01 (07)	01 (08)	02 (11)	01 (08)
Black or African European	02 (13)	01 (08)	00 (00)	00 (00)
White or Caucasian	12 (80)	09 (69)	15 (83)	12 (92)
Other	00 (00)	02 (15)	01 (06)	00 (00)
Time from infection to study enrollment §				
Recent (<6 months)	10 (67)	05 (38)	09 (50)	04 (31)
Long-term (>6 months)	05 (33)	07 (54)	07 (39)	08 (62)
Unknown	00 (00)	01 (08)	02 (11)	01 (08)
CD4+ T cell count - cells/mm^3	560 (252-1,497)	391 (286-1,333)	470 (203-930)	560 (230-1,098)
HIV-1 RNA level - copies/ml	39.207 (820-3,240,000)	49.400 (3,800-1,900,000)	67.550 (730-24,000,000)	41.000 (762-4,180,000)
HIV-1 subtype ¥				
B	07 (47)	06 (46)	08 (44)	07 (54)
Non-B	08 (53)	07 (54)	10 (56)	06 (46)
Human leukocyte antigen class I alleles ¥				
Risk: B*07, B*35	06 (40)	04 (31)	05 (28)	01 (08)
Protective: B*27, B*57, B*58	03 (20)	02 (15)	03 (17)	03 (23)

Data are median (range) or n (%)
§, Time from infection to study enrollment was self reported.
ART, antiretroviral therapy; RMD, romidepsin

Supplementary Table 2. Individual pre-ATI characteristics for the ATI population.

		HIV-1 subtype	HLA class I alleles						CD4+ T cells (per mm^3)	Intact HIV-1 proviruses (copies/10^6 CD4+ T cells)
			A1	A2	B1	B2	C1	C2		
ART (n=4)	120	B	02:03:01	02:03:01	46:01:01	51:01:02	01:02:01G	14:02:01G	930	891.00
	208	CRF other	02:02:01G	02:01:01G	14:02:01G	58:02:01G	06:02:01G	08:02:01G	843	104.10
	702	D	29:02:01G	66:01:01G	18:01:01G	35:01:01G	04:01:01G	05:01:01G	667	424.10
	707	CRF01	02:01:01G	02:01:01G	57:01:01G	57:01:01G	06:02:01G	06:02:01G	874	225.00
ART+3BNC117 (n=5)	109	D	11:01:01G	24:02:01G	07:02:01G	39:01:01G	07:02:01G	07:02:01G	900	167.90
	125	B	23:01:01G	26:01:01G	08:01:01G	40:01:01G	03:04:01G	07:01:01G	622	1141.00
	126	CRF01	02:06:01G	11:01:01G	15:21:01G	38:02:01G	04:03:01G	07:02:01G	710	7474.20
	703	CRF02	01:01:01G	24:02:01G	07:02:01G	08:01:01G	07:02:01G	07:01:01G	816	642.00
	704	CRF01	01:01:01G	24:02:01G	44:02:01G	57:01:01G	05:01:01G	06:02:01G	1,006	4121.60
ART+RMD (n=13)	104	B	02:01:01G	02:01:01G	15:01:01G	44:05:01G	02:02:02G	03:28	670	105.10
	117	B	02:01:01G	24:02:01G	40:02:01G	56:01:01G	01:02:01G	02:02:02G	880	19197.00
	204	C	03:01:01G	29:02:01G	07:02:01G	37:01:01G	06:02:01G	07:02:01G	576	198.70
	307	CRF01	01:01:01G	33:01:01G	14:02:01G	50:01:01G	06:02:01G	08:02:01G	610	2248.00
	708	CRF02	02:05:01G	24:02:01G	35:03:01G	41:01:01G	04:01:01G	17:01:01G	768	1815.00
ART+3BNC117 +RMD (n=16)	103	B	24:02:01G	26:01:01G	27:05:02G	38:01:01G	02:02:02G	12:03:01G	550	59.20
	107	CRF01	02:01:01G	25:01:01G	15:01:01G	44:02:01G	03:03:01G	05:03:01G	650	50.20
	112	G	02:01:01G	68:01:02G	15:78:01	44:02:01G	03:04:01G	07:04:01G	570	5148.40
	304	B	02:01:01G	02:01:01G	40:01:01G	51:01:01G	03:04:01G	15:02:01G	1,190	4044.00
	402	B	01:01:01G	23:01:01G	14:02:01G	45:01:01G	06:02:01G	08:02:01G	871	464.10
	709	B	01:01:01G	02:01:01G	15:01:01G	49:01:01G	03:04:01G	07:01:01G	652	44.60

ART, antiretroviral therapy; ATI, analytical treatment interruption; CRF, circulating recombinant form; HLA, human leukocyte antigen; RMD, romidepsin.

Supplementary Table 3. Example of the calculation of Gag-specific AIM$^+$ frequency for CD4$^+$ and CD8$^+$ T cells.

	CD4				
	41BB+CD69+PDL1+	41BB+CD69+PDL1-	41BB+CD69-PDL1+	41BB-CD69+PDL1+	
NEG	0.08%	0.13%	0.02%	0.63%	
GAG	0.16%	0.11%	0.06%	1.36%	GAG CD4 AIM+
GAG - NEG	0.08%	-0.02%	0.05%	0.73%	0.86%

	CD8				
	41BB+CD69+PDL1+	41BB+CD69+PDL1-	41BB+CD69-PDL1+	41BB-CD69+PDL1+	
NEG	0.12%	0.47%	0.02%	0.42%	
GAG	1.29%	0.92%	0.11%	1.05%	GAG CD8 AIM+
GAG - NEG	1.17%	0.45%	0.09%	0.63%	2.34%
