# Supplementary material for: Administration of broadly neutralizing anti-HIV-1 antibodies at ART initiation maintains long-term CD8+ T cell immunity
Source: Nat Commun. 2022 Oct 29;13:6473. doi: 10.1038/s41467-022-34171-2 (PMC9617872; doi:10.1038/s41467-022-34171-2)
Supplement: Supplementary file 2 — Reporting summary [file 41467_2022_34171_MOESM2_ESM.pdf]

nature portfolio

Corresponding author(s): Ole Schmeltz Søgaard

Last updated by author(s): October 11, 2022

Reporting Summary

Nature Portfolio wishes to improve the reproducibility of the work that we publish. This form provides structure for consistency and transparency in reporting. For further information on Nature Portfolio policies, see our Editorial Policies and the Editorial Policy Checklist.

Statistics

For all statistical analyses, confirm that the following items are present in the figure legend, table legend, main text, or Methods section.

n/a	Confirmed	
☐	☒	The exact sample size (*n*) for each experimental group/condition, given as a discrete number and unit of measurement
☐	☒	A statement on whether measurements were taken from distinct samples or whether the same sample was measured repeatedly
☐	☒	The statistical test(s) used AND whether they are one- or two-sided<br>*Only common tests should be described solely by name; describe more complex techniques in the Methods section.*
☐	☒	A description of all covariates tested
☐	☒	A description of any assumptions or corrections, such as tests of normality and adjustment for multiple comparisons
☐	☒	A full description of the statistical parameters including central tendency (e.g. means) or other basic estimates (e.g. regression coefficient) AND variation (e.g. standard deviation) or associated estimates of uncertainty (e.g. confidence intervals)
☐	☒	For null hypothesis testing, the test statistic (e.g. *F*, *t*, *r*) with confidence intervals, effect sizes, degrees of freedom and *P* value noted<br>*Give P values as exact values whenever suitable.*
☒	☐	For Bayesian analysis, information on the choice of priors and Markov chain Monte Carlo settings
☒	☐	For hierarchical and complex designs, identification of the appropriate level for tests and full reporting of outcomes
☒	☐	Estimates of effect sizes (e.g. Cohen's *d*, Pearson's *r*), indicating how they were calculated

Our web collection on statistics for biologists contains articles on many of the points above.

Software and code

Policy information about availability of computer code

Data collection	Study data were collected and managed in Research Electronic Data Capture (REDCap) electronic data capture tools version 11.1.29 hosted at the Clinical Trial Unit, Department of Clinical Medicine, Aarhus University, Aarhus, Denmark.
Data analysis	Flow Cytometry data were analyzed using FlowJo v10.7.2 for Mac. GraphPad Prism version 8.4.3 for Mac (San Diego, CA) was used for statistical analysis. Two-sided Mann-Whitney test and two-sided Wilcoxon matched paired test were used for unpaired and paired comparisons, respectively. P-values < 0.05 were considered significant. The analyses performed on primary and secondary endpoints were prespecified in the protocol, and no exploratory analyses were done, hence no corrections for multiple comparisons were made.

For manuscripts utilizing custom algorithms or software that are central to the research but not yet described in published literature, software must be made available to editors and reviewers. We strongly encourage code deposition in a community repository (e.g. GitHub). See the Nature Portfolio guidelines for submitting code & software for further information.

Data

Policy information about availability of data

All manuscripts must include a data availability statement. This statement should provide the following information, where applicable:

- Accession codes, unique identifiers, or web links for publicly available datasets
- A description of any restrictions on data availability
- For clinical datasets or third party data, please ensure that the statement adheres to our policy

Clinical data are not available for download due to privacy/ethical restrictions under the EU GDPR. Specific requests for access to the trial data may be sent to olesoega@rm.dk and access may be provided to a named individual in agreement with the rules and regulations of the Danish Data Protection agency and the

National Committee on Health Research Ethics. Source experimental data are provided with this paper. The sequences generated in this study have been deposited in the GenBank database under accession codes OP031483-OP031595.

Field-specific reporting

Please select the one below that is the best fit for your research. If you are not sure, read the appropriate sections before making your selection.

☒ Life sciences ☐ Behavioural & social sciences ☐ Ecological, evolutionary & environmental sciences

For a reference copy of the document with all sections, see nature.com/documents/nr-reporting-summary-flat.pdf

Life sciences study design

All studies must disclose on these points even when the disclosure is negative.

Sample size
All the samples available from all the 55 participants that received random allocation were tested. The sample size for each experiment is stated in the figure legend.

Data exclusions
For the AIM assay, data was excluded when the viability was lower than the pre-established threslhold of 80%.
For the IFN-g ELISpot, the pass/fail criteria set stated that the average of the negative wells (only cells) should have less than 20 spots per well. For positive controls (PHA) there should be greater than 100 spots per well. If any of these criteria were not met, then the plate was considered to have failed and data was not included.

Replication
This study was a clinical trial and the analyses were performed on individual trial participants. All available data is included in the manuscript.
Duplicates were performed in the ELISpot assay, being the median the final result presented in the manuscript.
Triplicates were performed in the viral inhibition assay , being the median the final result presented in the manuscript.

Randomization
The Clinical Trial Unit at Aarhus University generated the randomization sequence using permuted blocks of 4 or 8 by computer-generated random numbers. Randomization assignment was provided to each site through using REDCap.

Blinding
This study was open label.

Reporting for specific materials, systems and methods

We require information from authors about some types of materials, experimental systems and methods used in many studies. Here, indicate whether each material, system or method listed is relevant to your study. If you are not sure if a list item applies to your research, read the appropriate section before selecting a response.

Materials & experimental systems

n/a	Involved in the study	
☐	☒	Antibodies
☒	☐	Eukaryotic cell lines
☒	☐	Palaeontology and archaeology
☒	☐	Animals and other organisms
☐	☒	Human research participants
☐	☒	Clinical data
☒	☐	Dual use research of concern

Methods

n/a	Involved in the study	
☒	☐	ChIP-seq
☐	☒	Flow cytometry
☒	☐	MRI-based neuroimaging

Antibodies

Antibodies used
3BNC117 is an investigational anti-HIV-1 neutralizing antibody manufactured for clinical use. 3BNC117 is being investigated under US FDA IND 118225.
Antibodies used for the assessment of HIV-1-specific CD8+ T cell immunity:
Near IR Live Dead (Invitogen, L10119)
CD3 (PerCP/Cy5.5 anti-human CD3, SK7, BioLegend)
CD4 (BV650 anti-human CD4, RPA-T4, BioLegend)
CD8 (BV605 anti-human CD8a, RPA-T8, BioLegend)
4-1BB (PE anti-human CD137, BioLegend)
CD69 (APC anti-human CD69, FN50, BioLegend)
PD-L1 (BV421 anti-human CD274, B7-H1, BioLegend).

Validation
3BNC117 that was administered to the participants was manufactured by Celldex Therapeutics under Good Manufacturing Practice and has been fully characterized in terms of biophysical properties and potency (IND 118225). 3BNC117 is under long term stability monitoring.

Near IR Live Dead https://www.thermofisher.com/order/catalog/product/L10119
CD3 https://www.biolegend.com/en-us/products/percp-cyanine5-5-anti-human-cd3-antibody-6932
CD4 https://www.biolegend.com/en-us/products/brilliant-violet-650-anti-human-cd4-antibody-7650
CD8 https://www.biolegend.com/en-us/products/brilliant-violet-605-anti-human-cd8a-antibody-7651
4-1BB https://www.biolegend.com/en-us/products/pe-anti-human-cd137-4-1bb-antibody-1510
CD69 https://www.biolegend.com/en-us/products/apc-anti-human-cd69-antibody-1674
PD-L1 https://www.biolegend.com/en-us/products/brilliant-violet-421-anti-human-cd274-b7-h1-pd-l1-antibody-7261

Human research participants

Policy information about studies involving human research participants

Population characteristics

Newly-diagnosed ART naïve participants aged 18-65 years with a confirmed HIV-1 diagnosis and a CD4+ T cell count >200 cells/mm3 at screening were recruited by study physicians
Study participants were mainly Caucasian males and the median age was 36 (interquartile range [IQR], 28 47 years).

Recruitment

This was a phase 1b/2a, open-label, multicenter, randomized controlled trial enrolling at five sites in Denmark and two sites in the United Kingdom.
Our study may not be generalizable to all newly diagnosed individuals due to the study's stringent exclusion criteria.

Ethics oversight

The protocol was approved by the Danish Medicine Authorities in Denmark and the Medicines (#2016053184) and Healthcare Products Regulatory Agency (MHRA) in the United Kingdom (#31883/0001/001-0001), and the National Commitee on Health Research Ethics in Denmark (#1-10-72-110-16) and National Health Authority in the United Kingdom (#241439)
The Good Clinical Practice units located in Copenhagen, Aalborg, Aarhus and Odense monitored the study (https://gcp-enhed.dk/). Before any study-related procedures, written informed consent was obtained from the participant.

Note that full information on the approval of the study protocol must also be provided in the manuscript.

Clinical data

Policy information about clinical studies
All manuscripts should comply with the ICMJE guidelines for publication of clinical research and a completed CONSORT checklist must be included with all submissions.

Clinical trial registration

Clinicaltrial.gov: NCT03041012, EudraCT number: 2015-002234-53.

Study protocol

eCLEAR-001, version 3.1, 18 September 2018

Data collection

Eligible individuals were recruited from 16 January 2017 to 03 March 2020, and the last follow-up visit occurred on 17 July 2021.

Outcomes

The primary endpoints were 1) viral decay kinetics after ART initiation using plasma HIV-1 levels; and 2) changes in reservoir size measured by double-positive HIV-1 proviruses and HIV-Ag producing cells per million unfractionated CD4+ T cells. Secondary endpoints were 1) safety including CD4+ T cell counts; 2) changes in the transcriptionally and/or translationally active HIV-1-infected cells during the first 30 days of ART using FISH-flow assay 3) effects on HIV-1 Gag-specific T-cell immunity using the AIM assay; 4) time to loss of virologic control during ATI. We defined loss of virologic control as two consecutive plasma HIV-1 RNA measurements of ≥5,000 copies/ml. If CD4+ T-cell counts decreased to <350 cells/mm3, ART was also resumed18. 5) post-hoc baseline (day 0) sensitivity to 3BNC117 using PhenoSense or HIV-1 env sequencing and recency testing using the Asanté™ HIV-1 Rapid Recency® Assay.

Flow Cytometry

Plots

Confirm that:

☒ The axis labels state the marker and fluorochrome used (e.g. CD4-FITC).

☒ The axis scales are clearly visible. Include numbers along axes only for bottom left plot of group (a 'group' is an analysis of identical markers).

☒ All plots are contour plots with outliers or pseudocolor plots.

☒ A numerical value for number of cells or percentage (with statistics) is provided.

Methodology

Sample preparation

Cryopreserved PBMCs were thawed, washed and rested at 37C for 3 hours. Cells were then plated into wells of 96-well plate, at a total of 1×10^6 cells per well and stimulated with the different conditions. Following 20 hours of incubation at 37C cells were washed with PBS and stained for viability for 20 minutes. Cells were then incubated with TruStain FcX in PBS 2% FBS for 10 minutes and stained 30 minutes with surface markers antibodies. Cells were washed twice and acquired.

Instrument

All the samples were analyzed on a MACSQuant16 Flow Cytometer (Miltenyi Biotec)

Software	Flow cytometry data were analyzed using FlowJo software, version 10.6.0 (Tree Star).
Cell population abundance	No sorting was performed.
Gating strategy	Live cells > single cells > lymphocytes > CD3+ cells > CD4+ and CD8+ cells. AIM+ cells were defined as the frequency of cells that were either CD69+PD-L1+4-1BB+, CD69+PD-L1+, CD69+4-1BB+ or PD-L1+4-1BB+

☒ Tick this box to confirm that a figure exemplifying the gating strategy is provided in the Supplementary Information.
